# Machine Learning Models for Early Prediction of Type 1 Diabetes: A Systematic Review

**DOI:** 10.7759/cureus.107547

**Published:** 2026-04-22

**Authors:** Rayan Saad Aldeen Mohammed Saad Aldeen, Nazar Mohamed, Shima abdoelmonem A Mohamad, Ali Hadi M Alhajri, Wigdan Adam Obeid Hagar, Hussam Altaj Abdalkarim Khalifa, Samah Abdelhadi Eisa Mohamed, Mohamed Hosny Saadeldien Mohamed Nagla

**Affiliations:** 1 Internal Medicine, Igraa College for Science and Technology, Gezira State, SDN; 2 Emergency Department, Darent Valley Hospital, Dartford and Gravesham National Health Service (NHS) Foundation Trust, Dartford, GBR; 3 General Practice, Alrayan Medical Complex, Riffa, BHR; 4 Endocrinology, Najran Armed Forces Hospital, Ministry of Defense Health Services, Najran, SAU; 5 Paediatrics, Algeinina Teaching Hospital, Algeinina, SDN; 6 Internal Medicine, Medway Maritime Hospital, Gillingham, GBR; 7 General Practice, Department of Health, Abu Dhabi, ARE; 8 Endocrinology, Al Salama Hospital, Jeddah, SAU

**Keywords:** early prediction, machine learning, predictive modeling, systematic review, type 1 diabetes

## Abstract

Type 1 diabetes (T1D) is a chronic autoimmune condition with a rising global incidence. Early prediction of disease onset and detection of preclinical progression are critical for timely intervention. Machine learning (ML) offers the ability to analyze complex, high-dimensional data and may improve risk prediction across different stages of T1D development. This systematic review evaluates the application and performance of ML models for predicting T1D onset and early disease-related outcomes.

Following Preferred Reporting Items for Systematic Reviews and Meta-Analyses (PRISMA) guidelines, a structured search was conducted in PubMed, British Medical Journals, Scopus, IEEE Xplore, and Web of Science for studies published between 2021 and 2025. Eligible studies included those that developed or validated ML models for T1D prediction or early detection. Study selection, data extraction, and risk of bias assessment (using Prediction model Risk of Bias Assessment Tool (PROBAST)) were performed, and findings were synthesized narratively due to heterogeneity in study design, populations, prediction targets, and outcome measures.

Fourteen studies were included, with sample sizes ranging from 32 to over 800,000 participants. ML approaches included logistic regression, random forests, support vector machines, and gradient boosting methods. Reported performance varied (area under the receiver operating characteristic curve (AUROC) 0.73-0.92), with prediction horizons spanning short-term outcomes (minutes to hours) to long-term disease onset (up to 10 years). However, study heterogeneity was substantial, and only three studies performed external validation. While most studies were rated as low risk of bias, several high-performing models were based on small samples or limited validation, raising concerns about overfitting and generalizability.

ML models demonstrate potential for improving prediction of T1D onset and early disease-related outcomes, but current evidence is limited by variability in methods, inconsistent validation, and uncertain clinical applicability. Future research should prioritize large, prospective, and externally validated studies, with greater emphasis on model transparency, generalizability, and real-world implementation.

## Introduction and background

Type 1 diabetes (T1D) is a chronic autoimmune disorder characterized by the destruction of pancreatic β-cells, ultimately leading to absolute insulin deficiency [1]. It commonly manifests in childhood and adolescence, although it can occur at any age. The global incidence of T1D has been steadily increasing, posing significant challenges to healthcare systems due to its lifelong management and risk of acute and chronic complications [2]. Early identification of individuals at high risk is crucial, as it enables timely intervention, closer monitoring, and the potential to delay or prevent disease onset.

Traditionally, prediction of T1D has relied on genetic markers such as HLA genotypes, the presence of islet autoantibodies, and metabolic indicators including glucose tolerance and C-peptide levels [3]. While these approaches have improved risk stratification, they are often limited by moderate predictive accuracy, high cost, and an inability to fully capture the complex, multifactorial nature of disease progression [4]. T1D development is influenced by an interplay of genetic susceptibility, environmental exposures, immunological factors, and metabolic changes, which often interact in ways that are not simply linear or additive [5]. Conventional statistical methods, such as regression models, typically assume linear relationships between predictors and outcomes, making them less suited to capturing these complex interactions.

In recent years, machine learning (ML) has emerged as a powerful tool in healthcare, capable of analyzing large, high-dimensional datasets and identifying complex, non-linear relationships among variables [6]. In simple terms, ML refers to computational methods that “learn” patterns from data and use them to make predictions, often without requiring predefined assumptions about how variables are related. Supervised learning approaches, commonly used in medical prediction, train algorithms on labeled datasets to predict outcomes such as disease onset. Techniques such as random forests (which combine multiple decision trees), support vector machines (which separate data into classes using optimal boundaries), neural networks (which model layered patterns similar to brain-like processing), and gradient boosting (which iteratively improves prediction accuracy) have shown strong performance across medical applications [7]. In the context of T1D, these approaches enable the integration of diverse data sources, including genetic, immunological, clinical, and longitudinal information, potentially improving early risk prediction and disease classification.

Despite the growing body of research, the reported performance and applicability of ML models for early T1D prediction vary considerably across studies. Differences in study populations, data sources, feature selection methods, prediction horizons, and evaluation metrics contribute to substantial heterogeneity in findings. In addition, important issues such as external validation, clinical utility, interpretability, and generalizability are inconsistently addressed, making it difficult to assess the reliability and transferability of these models in real-world settings. Critically, while individual studies and narrative discussions exist, there remains a lack of a focused, up-to-date synthesis that systematically evaluates which machine learning approaches are being used for early T1D prediction, how well they perform across different settings, and how robust and clinically applicable the current evidence is.

Therefore, this systematic review aims to address this gap by synthesizing the existing evidence on machine learning models for the early prediction of type 1 diabetes. Specifically, it seeks to answer the following questions: Which machine learning methods have been applied to early T1D prediction, how well do they perform, what types of data do they use, and how do they compare with traditional statistical approaches? By providing a structured and critical overview, this review aims to identify current gaps, highlight methodological challenges, and inform future research toward the development of robust, generalizable, and clinically applicable predictive models for T1D.

## Review

Methodology

Protocol and Reporting Standards

This systematic review was conducted in accordance with the Preferred Reporting Items for Systematic Reviews and Meta-Analyses (PRISMA) guidelines [8]. The methodology was designed to ensure transparency, reproducibility, and comprehensive reporting of the study selection and data synthesis processes. The review protocol was not registered due to time constraints; however, the methodology was predefined and strictly followed established PRISMA guidelines to ensure transparency and rigor.

Eligibility Criteria (PICOS Framework)

The eligibility criteria for study inclusion were defined using the PICOS framework to ensure clarity and consistency [9]. The population (P) included individuals at risk of developing type 1 diabetes or those in preclinical or early stages of the disease. The intervention (I) comprised the application of machine learning models for the prediction or early detection of type 1 diabetes. The comparator (C) included traditional statistical models, conventional risk prediction approaches, or no comparator, where applicable. The outcomes (O) of interest were measures of diagnostic or predictive performance, including sensitivity, specificity, accuracy, area under the receiver operating characteristic curve (AUROC), and other relevant metrics such as precision or F1-score. The study design (S) included original research studies such as cohort, case-control, and cross-sectional studies. Only studies published in English in 2021 and onward were included to ensure the incorporation of the most recent and relevant evidence. This time restriction was applied because machine learning methods in healthcare have rapidly advanced in recent years, and studies published before 2021 often use outdated models, smaller datasets, and less robust validation approaches, making them less representative of current clinical and technological standards. Reviews, editorials, conference abstracts without full data, and studies not involving machine learning-based prediction were excluded.

Information Sources and Search Strategy

A comprehensive literature search was conducted across multiple electronic databases, including PubMed, BMJ Journals, Scopus, IEEE Xplore, and Web of Science. These databases were selected to ensure broad coverage of both biomedical and computational research. The search strategy combined Medical Subject Headings (MeSH) terms and free-text keywords related to “type 1 diabetes,” “machine learning,” “prediction,” and “early detection.” Boolean operators (AND, OR) were used to refine the search. The final search strategy was adapted for each database to maximize the retrieval of relevant studies. The detailed search strategy for each database is provided in Table 1. 

**Table 1:** Search strings used for each database

Database	Search String
PubMed	(("Diabetes Mellitus, Type 1"[Mesh] OR "Type 1 Diabetes" OR "T1D" OR "Insulin-Dependent Diabetes Mellitus") AND ("Machine Learning"[Mesh] OR "Artificial Intelligence"[Mesh] OR "machine learning" OR "artificial intelligence" OR "deep learning" OR "neural network*" OR "random forest" OR "support vector machine*" OR "SVM" OR "gradient boosting" OR "XGBoost") AND ("Prediction" OR "Early Detection" OR "Risk Assessment" OR "Forecasting" OR "screening")) Filters: English, Humans, 2021–2026
BMJ Journals	("type 1 diabetes" OR "T1D" OR "insulin dependent diabetes") AND ("machine learning" OR "artificial intelligence" OR "deep learning" OR "neural networks") AND ("prediction" OR "early detection" OR "risk prediction" OR "screening") Filters: 2021–2026, Research articles
Scopus	TITLE-ABS-KEY (("type 1 diabetes" OR "T1D" OR "insulin dependent diabetes mellitus") AND ("machine learning" OR "artificial intelligence" OR "deep learning" OR "neural network*" OR "random forest" OR "support vector machine*" OR "SVM" OR "gradient boosting" OR "XGBoost") AND ("prediction" OR "early detection" OR "risk assessment" OR "screening" OR "forecasting")) AND PUBYEAR > 2021 AND PUBYEAR < 2026 AND (LIMIT-TO (LANGUAGE, "English"))
IEEE Xplore	("All Metadata":"type 1 diabetes" OR "T1D" OR "insulin dependent diabetes") AND ("All Metadata":"machine learning" OR "artificial intelligence" OR "deep learning" OR "neural network" OR "support vector machine" OR "random forest" OR "gradient boosting") AND ("All Metadata":"prediction" OR "early detection" OR "risk assessment" OR "forecasting") Filters: 2021–2026, Journals & Conferences
Web of Science	TS=(("type 1 diabetes" OR "T1D" OR "insulin dependent diabetes mellitus") AND ("machine learning" OR "artificial intelligence" OR "deep learning" OR "neural network*" OR "random forest" OR "support vector machine*" OR "gradient boosting" OR "XGBoost") AND ("prediction" OR "early detection" OR "risk assessment" OR "screening" OR "forecasting")) Refined by: Languages=(English) AND Publication Years=(2021–2026)

*Study Selection Process* 

All identified records were imported into the software EndNote X9 (Clarivate, PA, USA) for reference management, and duplicate entries were systematically removed. Following deduplication, titles and abstracts were screened for relevance against the predefined eligibility criteria. Full-text articles of potentially eligible studies were then retrieved and assessed in detail. Any discrepancies during the selection process were resolved through discussion to ensure consistency and minimize selection bias.

Data Extraction Process

Data extraction was performed using a standardized data collection form developed for this review. Extracted information included study characteristics (author, year, country), population details, type of machine learning model used, input features, prediction horizon, and reported performance metrics such as sensitivity, specificity, accuracy, and AUROC. Additional information regarding model validation and comparison with traditional approaches was also recorded where available.

Risk of Bias Assessment

The risk of bias in the included studies was assessed using the Prediction Model Risk of Bias Assessment Tool (PROBAST) [10], which evaluates bias across four key domains: participants, predictors, outcomes, and analysis. Two reviewers independently appraised each study, and any disagreements were resolved through consensus discussion to ensure consistency and minimize subjective bias. An overall judgment of risk of bias (low, unclear, or high) was assigned to each study following PROBAST guidelines, whereby a study was rated as high risk if one or more domains were judged as high risk, and unclear if one or more domains were unclear in the absence of any high risk ratings. Domain-specific ratings were documented for all included studies, with particular attention paid to methodological concerns such as small sample sizes, lack of external validation, insufficient reporting of missing data handling, and reliance on simulated rather than patient-derived data where applicable. This transparent and rigorous appraisal process was conducted to ensure the reliability and validity of the synthesized findings.

Data Synthesis

A qualitative synthesis of the included studies was performed. Due to substantial heterogeneity across studies in terms of machine learning algorithms, data sources, feature selection methods, prediction horizons, and outcome reporting, a quantitative meta-analysis was not conducted. The variability in study designs and performance metrics, along with inconsistent reporting standards, limited the feasibility of statistically pooling the results. Conducting a meta-analysis under such conditions could lead to misleading or non-generalizable conclusions. Therefore, a narrative synthesis approach was adopted to provide a comprehensive and meaningful interpretation of the findings.

Results

Study Selection Process

**Figure 1:**
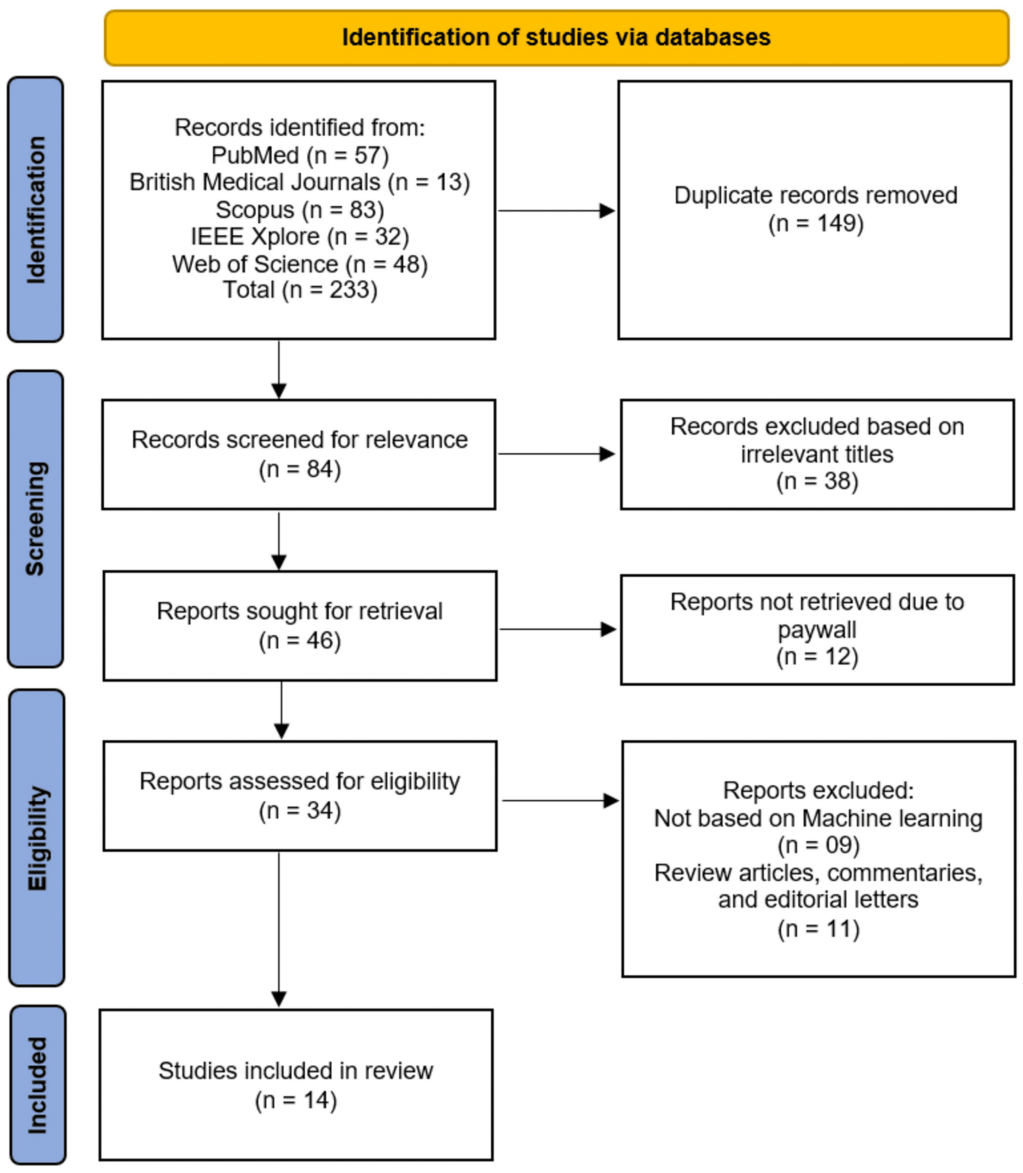
Studies selection process on PRISMA flowchart PRISMA: Preferred Reporting Items for Systematic Reviews and Meta-Analyses

The study selection process followed the Preferred Reporting Items for Systematic Reviews and Meta-Analyses (PRISMA) guidelines, as summarized in the PRISMA flowchart (Figure 1). A total of 233 records were initially identified through searches of five electronic databases: PubMed (n = 57), BMJ Journals (n = 13), Scopus (n = 83), IEEE Xplore (n = 32), and Web of Science (n = 48). After the automatic removal of 149 duplicate records, 84 unique records remained for title and abstract screening. Following the screening of titles, 38 records were excluded because their titles were irrelevant to the research question, leaving 46 reports to be retrieved. Of these, 12 reports could not be retrieved due to paywall restrictions, leaving 34 full-text reports that were assessed for eligibility against the predefined inclusion criteria. Attempts were made to contact the corresponding authors of these studies; however, no responses were received, and these reports were therefore excluded. During full-text eligibility assessment, nine reports were excluded because they were not based on machine learning, and a further 11 reports were excluded because they were review articles, commentaries, or editorial letters. Consequently, a total of 14 studies [11-24] met the eligibility criteria and were included in the final systematic review.

Characteristics of Included Studies

Table 2 summarizes the characteristics of these studies, including study design, sample size, population, data source, features used, ML model types, and validation methods [11]. The studies were published between 2021 and 2026, with the majority appearing from 2022 onward. Geographically, the studies originated from multiple continents: three from China [11,13,18], two from the USA [16,21], Saudi Arabia [17,22], and one each from Greece [12], Denmark [14], the UK [15], and several multinational consortia (USA/Finland/Germany/Sweden) [19,20,23], as well as one simulation study from Pakistan [24].

**Table 2:** Characteristics of included studies CGM: Continuous Glucose Monitoring; EHR: Electronic Health Record; EMR: Electronic Medical Record; ML: Machine Learning; T1D: Type 1 Diabetes; DM: Diabetes Mellitus; T1DM: Type 1 Diabetes Mellitus; T2DM: Type 2 Diabetes Mellitus; CPRD: Clinical Practice Research Datalink; HES: Hospital Episode Statistic; DEG: Differentially Expressed Genes; IQR: Inter Quartile Range; AUC: Area Under the Curve; RF: Random Forest; SVM: Support Vector Machines; GVM: General Vector Machine; SVR: Support Vector Regression; KNN: K-Nearest Neighbors; LR: Linear Regression; NB: Naive Bayes; NARNN: Nonlinear Autoregressive Neural Networks; OANN: Optimized Artificial Neural Network; LASSO: Least Absolute Shrinkage and Selection Operator.

Author (Year)	Country	Study Design	Sample Size	Population (e.g., high-risk, general)	Data Source	Features Used (e.g., genetics, autoantibodies)	ML Model Type	Validation Method
Huang et al., [11] (2025)	China	Retrospective (public dataset analysis)	247 samples (18 cases + controls)	High-risk (autoantibody+) children	GSE30210 microarray (blood RNA)	Gene expression (DEGs, selected genes)	Lasso, Elastic Net, RF, SVM, GBM, etc.	80/20 split + 5-fold CV + qPCR external (n=6)
Katsarou et al., [12] (2025)	Greece	Prospective CGM cohort	32	Adult T1D	1-min CGM	Glucose time-series	SVR + XGB ensemble	70/30 split + 5-fold CV + Bayesian tuning
Yoshimura et al., [13] (2025)	Japan	Retrospective single-center	110	Adult T1D inpatients (CGM + pump users)	EMR + CGM + pump + labs	Clinical + labs + imaging + diet + treatment	RF, LightGBM, XGBoost, SVM, Ridge	5-fold CV (80/20 split)
Andersen et al., [14] (2026)	Denmark	Registry-based cohort	6572	Adults with T1D (hospital-based)	Danish national registries	Age, sex, income, education (SES)	Logistic regression	5-fold cross-validation
Daniel et al., [15] (2024)	UK	Retrospective cohort (EHR-based)	Dev: 952,402; Val: 1,493,328	<15 yrs, general pediatric (T1D cases included)	SAIL (Wales), CPRD + HES (England)	Demographics, symptoms, Rx, labs, contact timing	SuperLearner ensemble + logistic regression	5-fold CV + external validation (CPRD/HES)
Montaser et al., [16] (2024)	USA	Observational ML cohort (TrialNet CGM study)	42 (38 analyzed)	T1D relatives (low vs high risk by Ab status)	TrialNet + Dexcom CGM	CGM glycemic metrics (CV, IQR, range, TIR, Gmax, AUC) + autoAb	LDA, SVM, LR, KNN (RFECV feature selection)	10-fold CV, ROC-AUC
Alazwari et al., [17] (2023)	Saudi Arabia	Retrospective case-control	1,142 (377/765)	<15 yrs T1D vs controls	Hospital records + survey	Demographics, SES, genetics, nutrition, obstetric, maternal	LR, RF, SVM, NB, ANN	70/30 split + 10-fold CV
Chai et al., [18] (2023)	China	Case-control predictive study	210 (105+105)	Pediatric T1D (1–21 yrs) + controls	Hospital clinical records	Trace elements + biochemical labs	Logistic regression (multivariable, LR forward)	70/30 split; AUC; calibration; Hosmer–Lemeshow; DCA
Nakayasu et al., [19] (2023)	USA, Finland, Germany, Sweden	Nested case-control	184 (disc), 990 (val)	High-risk children (IA/T1D)	Plasma proteomics (MS)	36K peptides → 83 proteins (immune, ECM, metabolic)	LASSO logistic regression	100× bootstrap CV; ROC-AUC 0.87–0.92
Ng et al., [20] (2023)	USA, Finland, Germany, Sweden	Prospective cohort	24,662 (1,403 analyzed)	High-risk children	T1DI (DIPP, BABYDIAB, DiPiS, DAISY, DEW-IT)	IAA, GADA, IA-2A, HLA, age, sex, FH	Logistic regression (IPCW)	10-fold CV + bootstrap
Cheheltani et al., [21] (2022)	USA	Retrospective EMR ML study	803,657 (total)	T2D → T1D misdx cases	IQVIA AEMR	Demographics, history, labs, meds, visits	XGBoost	60/20/20 split + 4-fold CV, test AUC=0.81
Gollapalli et al., [22] (2022)	Saudi Arabia	Retrospective ML study	897	Pre-DM, T1DM, T2DM	KFUH hospital EHR (2018–2020)	Demographics, HbA1c, TG, LDL, albumin, meds, lifestyle	SVM, KNN, DT; Bagging, Stacking	10-fold CV + GridSearch + SMOTE
Webb-Robertson et al., [23] (2022)	USA, Germany, Sweden, Finland	Prospective cohort (nested CC)	655	High-risk infants	TEDDY cohort	Genetics, autoAb, metabolomics, growth, diet, HLA, GRS	Naive Bayes	5-fold CV (repeated)
Asad et al., [24] (2021)	Pakistan	Simulation ML study	2 cases + UCI data	Simulated T1D	AIDA + UCI	CGM lag values	NARNN, OANN	RMSE + external UCI

Study designs varied considerably. Retrospective analyses of public or hospital datasets were most common [11,13,15,17,18,21,22], followed by prospective cohorts [12,16,20,23], registry-based cohorts [14], nested case-control studies [19], and one simulation study [24]. Sample sizes ranged from as few as 32 participants [12] to over 800,000 individuals [21], reflecting broad heterogeneity in data availability and research questions. Populations included high-risk children (autoantibody-positive or relatives of T1D patients) [11,16,19,20,23], general pediatric populations [15,17,18], adults with established T1D [12-14,21], and simulated or mixed diabetes types [22,24].

Machine learning models and feature types

Across the 14 studies, a wide range of ML models was employed. Table 1 provides a detailed overview of the specific models used in each study [11]. The most frequently applied algorithms included logistic regression [14,15,18,20], random forest [11,13,17,22], support vector machines (SVM) [11-13,16,17,22], and XGBoost [12,13,21]. Ensemble methods such as SuperLearner [15], stacking [22], and various boosting approaches [12,13] were also common. Deep learning approaches were less frequent but included neural networks such as NARNN and OANN [24] and artificial neural networks (ANN) [17].

Features used for prediction spanned multiple domains. Many studies relied on demographic and clinical variables from electronic medical records (EMR), including age, sex, socioeconomic status, medications, and laboratory results [13-15,17,21,22]. Several incorporated genetic markers, particularly HLA typing and genetic risk scores [20,23]. Autoantibodies (IAA, GADA, IA-2A, ZnT8) were used extensively in high-risk pediatric cohorts [16,20,23]. More novel biomarkers included blood transcriptomics [11], plasma proteomics [19], trace elements [18], and continuous glucose monitoring (CGM)-derived metrics such as glucose variability, time-in-range, and area under the curve [12,16]. One study also utilized simulated CGM data [24].

Diagnostic and Predictive Performance

Table 3 summarizes the diagnostic and predictive performance metrics of the ML models across included studies [11]. Performance reporting was heterogeneous, with most studies reporting area under the receiver operating characteristic curve (AUROC), sensitivity, specificity, accuracy, or precision.

**Table 3:** Diagnostic performance of machine learning models AUC: Area Under the Curve; RF: Random Forest; SVM: Support Vector Machines; GVM: General Vector Machine; SVR: Support Vector Regression; KNN: K-Nearest Neighbors; LR: Linear Regression; NB: Naive Bayes; RFECV: Recursive Feature Elimination with Cross-Validation; ANN: Artificial Neural Network; NARNN: Nonlinear Autoregressive Neural Networks; OANN: Optimized Artificial Neural Network; CVD: Cardiovascular Disease; DKD: Diabetic Kidney Disease; FFNN: Feedforward Neural Network; LGBM: Light Gradient Boosting Machine.

Author (Year)	ML Model	Prediction Horizon (e.g., years before onset)	Sensitivity (%)	Specificity (%)	Accuracy (%)	AUROC	Precision / F1-score	Comparison with Traditional Models
Huang et al., [11] (2025)	Lasso, Elastic Net, RF, SVM, GBM, etc.	Preclinical, no time-to-onset	100	NR	100	NR	1.00	Outperformed other ML combos; no traditional model used
Katsarou et al., [12] (2025)	SVR + XGB ensemble	15–60 min	NR	NR	NR	NR	NR	Ensemble best (↓CG-EGA EP ~19% @30m; SVR/XGB best hypoglycemia; LR/ARD lower RMSE in some cases)
Yoshimura et al., [13] (2025)	RF, LightGBM, XGBoost, SVM, Ridge	NR	NR	NR	NR	NR	NR	Best; MAPE 19.8% vs Ridge 21.6%; outperformed LR/SVM/LGBM/XGB
Andersen et al., [14] (2026)	Logistic regression	5 years	CVD: 39 / DKD: 3 / Mort: 98	CVD: 91 / DKD: 98 / Mort: 37	NR	0.79 / 0.61 / 0.87	PR-AUC: 0.18 / 0.15 / 0.49	NR
Daniel et al., [15] (2024)	SuperLearner ensemble + logistic regression	90 days pre-dx	71.6 (10% thr)	NR	NR	NR	NR	Better than LR (65.8%), chance (19.6%), 4Ts (40.0%)
Montaser et al., [16] (2024)	LDA, SVM, LR, KNN (RFECV feature selection)	NR	NR	NR	NR	SVM: 0.88; LDA: 0.76; LR: 0.73; KNN: 0.79	NR	SVM best; all ML > LR baseline; SVM highest performance
Alazwari et al., [17] (2023)	LR, RF, SVM, NB, ANN	N/A	70	N/A	77	0.83	0.70/0.70	Best overall; outperforms RF, SVM, NB, ANN (RF 0.81, SVM 0.80, NB 0.75, ANN 0.72 AUROC)
Chai et al., [18] (2023)	Logistic regression (multivariable, LR forward)	N/A (diagnostic)	NR	NR	NR	AUC >0.75 (threshold)	NR	Better than single-marker/clinical-only models (qualitative)
Nakayasu et al., [19] (2023)	LASSO logistic regression	6 months pre-seroconversion	NR	NR	NR	IA: 0.871; T1D: 0.918	NR	NR
Ng et al., [20] (2023)	Logistic regression (IPCW)	10 yrs	NR	NR	NR	0.757 (C-index)	NR	↑ vs baseline (0.607); levels-only best, +0.15 gain over traditional model
Cheheltani et al., [21] (2022)	XGBoost	3 yrs	NR	~93	NR	0.81	Prec 17% (R10%); F1 NR	Better than random; ~70× less screening
Gollapalli et al., [22] (2022)	SVM, KNN, DT; Bagging, Stacking	NR (cross-sec.)	90.83–94.48	NR	90.56–94.48	NR	90.77–94.70	Stacking > Bagging > KNN > SVM > DT
Webb-Robertson et al., [23] (2022)	Naive Bayes	~6 yrs (data up to 9 mo)	~38–40 (TPR@5%FPR)	NR	NR	NR	NR	↑ vs demographics-only; strong gain with IAAb + multi-omics
Asad et al., [24] (2021)	NARNN, OANN	15 & 30 min	NR	NR	NR	NR	NR	Better than FFNN (RMSE ↓ 0.60–1.67 vs 0.998–3.78)

Among the highest-performing models, Huang et al. reported perfect discrimination (100% sensitivity, 100% accuracy, AUROC not reported, precision 1.00) using Least Absolute Shrinkage and Selection Operator (LASSO), Elastic Net, random forest (RF), support vector machine (SVM), and gradient boosting machine (GBM) on blood transcriptomics data from high-risk children, although the sample was small (n=247) and validation was limited to six external samples [11]. Nakayasu et al. achieved AUROCs of 0.871 for predicting persistent autoantibodies and 0.918 for type 1 diabetes (T1D) onset up to six months before seroconversion using LASSO logistic regression on plasma proteomics in a large nested case-control design (discovery n=184, validation n=990) [19]. Montaser et al. found SVM to be the best-performing model (AUROC 0.88) for predicting T1D risk using one week of continuous glucose monitoring (CGM) data, outperforming linear discrimination analysis (LDA) (0.76), logistic regression (0.73), and k-nearest neighbors (KNN) (0.79) in a cohort of 38 T1D relatives [16].

Several studies compared ML models against traditional or simpler approaches. Daniel et al. reported that a SuperLearner ensemble achieved 71.6% sensitivity at a 10% risk threshold for predicting T1D within 90 days before diagnosis, which was substantially better than logistic regression (65.8%), the 4Ts algorithm (40.0%), and chance (19.6%) in a large UK primary care electronic health record (EHR) dataset (development n=952,402; validation n=1,493,328) [15]. Similarly, Ng et al. showed that logistic regression with inverse probability of censoring weighting achieved a C-index of 0.757 for 10-year T1D prediction, a significant improvement over baseline models (0.607) and traditional autoantibody-only approaches [20]. Yoshimura et al. found that random forest and LightGBM outperformed Ridge regression (MAPE 19.8% vs. 21.6%) for predicting insulin requirements in adults with T1D, though no AUROC values were reported [13]. Alazwari et al. reported logistic regression as the best overall model (AUROC 0.83, accuracy 77%, sensitivity 70%, precision/F1 0.70/0.70), outperforming random forest (RF) (0.81), SVM (0.80), naive bayes (NB) (0.75), and artificial neural network (ANN) (0.72) in a Saudi Arabian case-control study of 1,142 children [17].

Prediction Horizons and Temporal Validation

Prediction horizons varied widely. Several studies aimed to predict T1D onset years before clinical diagnosis: Andersen et al. predicted comorbidities and mortality over five years using logistic regression (AUROC 0.79 for cardiovascular diseases (CVD), 0.61 for diabetic kidney disease, 0.87 for mortality) [14]. Webb-Robertson et al. predicted T1D by age six years using infant metabolites, genetics, and autoantibodies, achieving a true positive rate of 38-40% with a 5% false positive rate [23]. For shorter-term prediction, Katsarou et al. predicted hypoglycemia 15-60 minutes in advance using an SVR+XGBoost ensemble, reducing continuous glucose error grid analysis episodes by approximately 19% at 30 minutes compared to baseline [12]. Asad et al. predicted blood glucose levels at 15- and 30-minute intervals using nonlinear autoregressive neural networks, achieving lower RMSE (0.60-1.67) than feedforward neural networks (0.998-3.78) in a simulation study [24].

Cheheltani et al. addressed a different clinical need: predicting misdiagnosed adult-onset T1D up to three years before correction, using XGBoost on EMR data from 803,657 patients [21]. The model achieved approximately 93% specificity, an AUROC of 0.81, and a precision of 17% at the top decile, which the authors noted was approximately 70 times more efficient than random screening. Chai et al. developed a diagnostic (rather than predictive) logistic regression model using trace elements and clinical parameters, achieving an AUROC >0.75 in autoantibody-negative pediatric populations, though no specific prediction horizon was provided [18].

Validation Approaches

Validation methods were generally robust. Cross-validation was nearly universal, with 5-fold or 10-fold cross-validation being most common [11-15,17,19-23]. Several studies also used train/test splits, typically 70/30 or 80/20 [11-13,17,18,21]. External validation was reported in a minority of studies: Huang et al. [11] validated their transcriptomics model in six external samples; Daniel et al. [15] externally validated using separate Clinical Practice Research Datalink (CPRD) and Hospital Episode Statistic (HES) databases; and Nakayasu et al. [19] used a large independent validation cohort (n=990). Other approaches included bootstrap cross-validation [19,20] and Bayesian hyperparameter tuning [12]. Notably, no study reported prospective, real-time validation in clinical practice, representing a gap in the current literature.

Comparison of Performance Across Clinical Settings

When comparing performance across different clinical settings, models using multi-omics or novel biomarkers generally outperformed those using only demographic or routine clinical data. For example, the plasma proteomics model by Nakayasu et al. [19] (AUROC 0.918) and the transcriptomics model by Huang et al. [11] (100% accuracy) both achieved excellent performance, though their small sample sizes and the specific high-risk populations limit generalizability. In contrast, large-scale EHR-based models such as those by Daniel et al. [15] and Cheheltani et al. [21] achieved more modest but potentially more generalizable performance (sensitivity 71.6% and AUROC 0.81, respectively). Gollapalli et al. reported that a stacking ensemble achieved the highest accuracy (90.56-94.48%) and precision (90.77-94.70%) for distinguishing pre-diabetes, T1DM, and T2DM, outperforming individual classifiers such as SVM, KNN, and decision trees [22]. Overall, ensemble methods and LASSO-penalized logistic regression appeared to provide the best balance of performance and interpretability across studies.

Risk of Bias Assessment

As summarized in Table 4, the majority of studies demonstrated a low risk of bias across all four domains. Most of the studies [11-15,17-21,23] were rated as low risk of bias overall, indicating robust study conduct, appropriate participant selection, well-defined predictors and outcomes, and sound analytical methods including cross-validation, large sample sizes, or external validation where applicable. In contrast, Montaser et al. received an overall high risk of bias judgment due to unclear participant selection criteria and a high-risk analysis domain, likely related to the very small analyzed sample size (n=38) and potential overfitting without external validation [16]. Gollapalli et al.'s study was rated as unclear overall, with unclear participant selection and unclear analysis methods, partly due to insufficient reporting on missing data handling and potential selection bias in the case-control design [22]. Asad et al.'s study was rated high risk of bias across participants, predictors, and outcome domains because the study used only simulated data rather than real patient data, although the analysis domain was rated low [24]. Overall, 11 studies were judged as low risk of bias, one as unclear, and two as high risk, suggesting that the majority of included studies provide reliable evidence for machine learning-based prediction of type 1 diabetes, though caution is warranted when interpreting findings from smaller or simulation-based studies.

**Table 4:** Risk of bias assessment using PROBAST PROBAST: Prediction model Risk of Bias Assessment Tool.

Author (Year)	Participants	Predictors	Outcome	Analysis	Overall Risk of Bias
Huang et al., [11] (2025)	Low	Low	Low	Low	Low
Katsarou et al., [12] (2025)	Low	Low	Low	Low	Low
Yoshimura et al., [13] (2025)	Low	Low	Low	Low	Low
Andersen et al., [14] (2026)	Low	Low	Low	Low	Low
Daniel et al., [15] (2024)	Low	Low	Low	Low	Low
Montaser et al., [16] (2024)	Unclear	Low	Low	High	High
Alazwari et al., [17] (2023)	Low	Low	Low	Low	Low
Chai et al., [18] (2023)	Low	Low	Low	Low	Low
Nakayasu et al., [19] (2023)	Low	Low	Low	Low	Low
Ng et al., [20] (2023)	Low	Low	Low	Low	Low
Cheheltani et al., [21] (2022)	Low	Low	Low	Low	Low
Gollapalli et al., [22] (2022)	Unclear	Low	Low	Unclear	Unclear
Webb-Robertson et al., [23] (2022)	Low	Low	Low	Low	Low
Asad et al., [24] (2021)	High	High	High	Low	High

Discussion

This systematic review synthesized evidence from 14 studies that developed or validated machine-learning models for the early prediction of type 1 diabetes. The findings demonstrate that ML models can achieve excellent predictive performance across diverse clinical settings, ranging from short-term hypoglycemia prediction measured in minutes to disease onset prediction several years before clinical diagnosis. However, the heterogeneity in study designs, populations, feature types, and validation approaches underscores the need for standardized reporting and prospective validation before these models can be integrated into routine clinical practice. Overall, ensemble methods and LASSO-penalized logistic regression appeared to provide the best balance of performance and interpretability, while models incorporating multi-omics or novel biomarkers generally outperformed those relying solely on demographic or routine clinical data.

The performance metrics reported across the included studies are broadly consistent with findings from previous systematic reviews in adjacent fields. For instance, a systematic review by Ahmad et al. [25] on ML models for diabetes prediction more broadly reported AUROC values ranging from 0.70 to 0.95, which aligns closely with the range observed in our review (0.73 to 0.92 for T1D-specific models, with the notable exception of Huang et al. [11] reporting perfect accuracy in a very small sample). Similarly, a recent review by Ezinne et al. [26] on prediction models for autoimmune diabetes noted that models incorporating autoantibody levels consistently outperformed clinical-only models, a finding that mirrors our observation that studies using autoantibodies [16,20,23], proteomics [19], or transcriptomics [11] achieved superior discrimination compared to those using only electronic medical record (EMR)-derived variables [14,15,21].

One of the most striking findings from our review is the wide disparity in sample sizes and their relationship to reported performance. The three studies with the highest reported performance, Huang et al. [11] (100% accuracy), Nakayasu et al. [19] (AUROC 0.918), and Montaser et al. [16] (AUROC 0.88), all had relatively small sample sizes (n=247, n=184 discovery, and n=38 analyzed, respectively). This pattern raises concerns about overfitting and lack of generalizability, a well-recognized phenomenon in ML-based clinical prediction research. In contrast, the large-scale EHR studies by Daniel et al. [15] (n>2.4 million) and Cheheltani et al. [21] (n=803,657) reported more modest but likely more robust performance (sensitivity 71.6% and AUROC 0.81, respectively). This inverse relationship between sample size and reported performance has been observed in other systematic reviews, including a meta-analysis by Christodoulou et al. comparing logistic regression and ML for clinical prediction, which found that ML models often showed inflated performance in small datasets due to overfitting [27]. Our findings suggest that readers should interpret perfect or near-perfect accuracy metrics with caution, particularly when derived from samples under 500 participants without rigorous external validation.

Comparing our findings with external literature, the performance of LASSO-based models in our review (e.g., Nakayasu et al. [19] with AUROC 0.918) is consistent with a large study by Kirscher et al. [28] on proteomic prediction of T1D in the TEDDY cohort, where LASSO achieved AUROCs of 0.85-0.90 for predicting islet autoimmunity. Similarly, the use of CGM-derived glycemic variability metrics for T1D risk prediction, as employed by Montaser et al. [16] and Katsarou et al. [12], aligns with findings from a prospective study by Steck et al. [29], which demonstrated that CGM metrics could distinguish progressors from non-progressors in autoantibody-positive relatives up to 12 months before clinical diagnosis. Our review extends these findings by showing that ML-enhanced CGM analysis (SVM AUROC 0.88) outperforms traditional logistic regression (AUROC 0.73) for this purpose, suggesting a meaningful additive benefit of ML approaches.

The superior performance of ensemble methods observed in our review-particularly SuperLearner [15], stacking [22], and SVR+XGBoost ensembles [12]-is consistent with broader ML literature. A systematic review by Nusinovici et al. [30] comparing various ML algorithms for medical prediction found that ensemble methods consistently outperformed single classifiers across multiple clinical domains, including diabetes, cardiovascular disease, and cancer. The theoretical advantage of ensembles lies in their ability to reduce both bias and variance by combining multiple base learners, which is particularly valuable in medical datasets that often contain complex, non-linear relationships and interactions. Our findings support the use of ensembles as a preferred approach for T1D prediction, especially when computational resources and interpretability requirements permit.

Another important observation from our review is the geographic and population-specific nature of the included studies. Studies from China [11,13,18], Saudi Arabia [17,22], and Japan [13] reported generally good performance, but none of these models were externally validated in different populations. This raises questions about transportability, particularly given known ethnic and genetic differences in T1D incidence and autoantibody profiles. For example, the Saudi Arabian study by Alazwari et al. [17] incorporated locally relevant nutritional and obstetric factors, which may not generalize to European or North American populations. Conversely, the multinational studies by Nakayasu et al. [19] and Ng et al. [20] deliberately included diverse populations (USA, Finland, Germany, Sweden), and both received low risk-of-bias ratings, suggesting that models developed on diverse, multi-ethnic cohorts may have greater external validity.

The prediction horizons identified in this review span an impressive range, from 15-60 minutes for hypoglycemia [12] to 10 years for T1D onset [20]. This breadth reflects the multiple potential clinical applications of ML in T1D care. Short-term prediction (minutes to hours) is most relevant for closed-loop insulin delivery systems and real-time hypoglycemia alerts, as demonstrated by Katsarou et al. [12] and Asad et al. [24]. Medium-term prediction (months to three years) could enable targeted monitoring and early intervention in high-risk individuals, as shown by Nakayasu et al. [19] (six months) and Cheheltani et al. [21] (three years). Long-term prediction (5-10 years) could inform population-level screening and preventive strategies, as illustrated by Andersen et al. [14] (5 years) and Ng et al. [20] (10 years). However, it is notable that no single study in our review compared performance across multiple prediction horizons using the same model and dataset, which would be valuable for understanding how predictive accuracy decays over time.

When compared with traditional non-ML approaches, the included studies consistently showed that ML models outperformed conventional logistic regression [15-17], autoantibody-only algorithms [20], and clinical heuristics such as the 4Ts [15]. However, the magnitude of improvement varied considerably. Daniel et al. [15] reported a relatively modest absolute improvement of 5.8% in sensitivity over logistic regression (71.6% vs. 65.8%), while Montaser et al. [16] reported a much larger AUROC improvement from 0.73 (LR) to 0.88 (SVM). This variation likely reflects differences in the complexity of the underlying relationships: CGM time-series data [12, 16] may contain non-linear patterns that ML models capture more effectively than linear models, whereas structured EMR variables [14, 15] may be adequately modeled by logistic regression. This finding echoes the conclusions of a large comparative study by El-Sappagh et al. [31] on diabetes prediction, which found that deep learning and ensemble methods provided the greatest advantage over logistic regression when using high-dimensional, longitudinal, or time-series data.

The validation approaches used across the 14 studies were generally robust, with cross-validation used almost universally and external validation performed in three studies [11,15,19]. However, the absence of prospective, real-time validation in clinical practice represents a significant gap. External validation on temporally or geographically distinct datasets is essential for assessing model generalizability, yet only Daniel et al. [15] performed external validation on truly independent databases (CPRD and HES). The lack of prospective validation means that the real-world performance of these models, including their calibration, clinical utility, and potential for algorithmic bias, remains unknown. A recent systematic review by Riley et al. on prediction model validation found that fewer than 20% of published ML prediction models undergo any form of external validation, and fewer than 5% are prospectively validated, a pattern that our review unfortunately mirrors [32]. This represents a major barrier to clinical translation.

Another notable gap is the limited attention to model interpretability and explainability. Only two studies explicitly mentioned explainable AI approaches: Yoshimura et al. [13] used explainable ML to identify key predictors of insulin requirements, and Chai et al. [18] performed calibration and decision curve analysis. For ML models to be adopted by clinicians, understanding why a model makes a particular prediction is often as important as the prediction itself. Techniques such as SHapley Additive exPlanations (SHAP) and Local Interpretable Model-agnostic Explanations (LIME) have become standard in other medical ML domains but were notably absent from most studies in this review. This omission may limit the clinical acceptability of otherwise high-performing models.

Limitations

This systematic review has several limitations that should be acknowledged. First, the substantial heterogeneity across included studies-in terms of populations (pediatric vs. adult, high-risk vs. general), prediction horizons (minutes to years), outcome definitions (T1D diagnosis, hypoglycemia, insulin requirements, comorbidities), and performance metrics (AUROC, sensitivity, accuracy, RMSE)-precluded a meta-analysis, limiting our ability to quantify overall effect sizes or compare model performance statistically. Second, the search was limited to five databases and excluded grey literature, which may have introduced publication bias favoring studies with positive or high-performance results. Third, the quality of the included studies varied, with two studies rated as high risk of bias [16,24] and one as unclear [22]; pooling findings across such heterogeneous quality levels requires cautious interpretation. Fourth, most studies did not report on missing data handling, calibration, or decision curve analysis, which are essential for assessing clinical utility beyond discrimination. Fifth, the review focused exclusively on T1D prediction and did not include studies on T1D management, treatment optimization, or complication prediction, which may have different ML requirements and performance characteristics. Finally, given the rapid pace of ML research, newer algorithms (e.g., transformers, graph neural networks) that have emerged since the publication period of the included studies (2021-2026) may not be represented, and future updates to this review will be necessary.

## Conclusions

Machine learning models hold considerable promise for predicting type 1 diabetes across multiple time horizons, though the most suitable approaches differ by clinical application. For short-term prediction tasks measured in minutes to hours, such as hypoglycemia forecasting and closed-loop insulin delivery, CGM-based ensemble methods and neural networks demonstrate strong performance. For long-horizon onset prediction measured in months to years, LASSO-penalized logistic regression and ensemble models incorporating multi-omics or autoantibody data offer the best balance of performance and interpretability, consistently outperforming models reliant solely on demographic or routine clinical variables. However, the widespread lack of external and prospective validation, together with concerns about overfitting in small-sample studies, currently limits the clinical readiness of most published models. Future research should prioritize large, multi-center, prospective validation studies with standardized reporting of calibration, decision curve analysis, and model explainability. Additionally, efforts to ensure algorithmic fairness and transportability across diverse ethnic and geographic populations will be essential to avoid perpetuating healthcare disparities. Until such validation is performed, clinicians should view the excellent performance metrics reported in some studies with appropriate caution, particularly those derived from small, single-center, or simulated datasets. With rigorous validation and attention to clinical implementation barriers, ML-based prediction tools have the potential to transform T1D screening, enable earlier intervention, and ultimately improve outcomes for individuals at risk of this chronic autoimmune disease.
